# Occurrence of Human Enteric Viruses in Retail Mussels Marketed in Burgos, Spain

**DOI:** 10.3390/microorganisms14092099

**Published:** 2026-09-19

**Authors:** Mariana Alves Elois, Nadine Yeramian, Daniel Perez-Alonso, Rafael Dorighello Cadamuro, Antonio Valero-Díaz, Álvaro Cañete-Reyes, Henrique Borges da Silva, Gislaine Fongaro, David Rodríguez-Lázaro

**Affiliations:** 1 Laboratory of Applied Virology, Department of Microbiology, Immunology, and Parasitology, Federal University of Santa Catarina, Florianópolis 88035-972, Brazil; malves@ubu.es (M.A.E.); rdorighello@ubu.es (R.D.C.); hgrisard@gmail.com (H.B.d.S.); gislaine.fongaro@ufsc.br (G.F.); 2 Laboratory of Microbiology, Department of Biotechnology and Food Science, University of Burgos, 09001 Burgos, Spain; nyeramian@ubu.es (N.Y.); dperez@ubu.es (D.P.-A.); acanete@ubu.es (Á.C.-R.); 3 Centre for Emerging Pathogens and Global Health, University of Burgos, 09001 Burgos, Spain; 4 Department of Food Science and Technology, UIC Zoonosis y Enfermedades Emergentes (ENZOEM), University of Cordoba, 14014 Cordoba, Spain; bt2vadia@uco.es

**Keywords:** bivalve mollusks, norovirus, hepatitis A virus, hepatitis E virus, RT-qPCR, food safety, viral surveillance

## Abstract

Bivalve mollusks are recognized as bioaccumulators of enteric viruses and, therefore, represent important environmental sentinels for microbiological contamination. The contamination of these organisms is primarily associated with the exposure of coastal waters to human and agricultural effluents, which introduce pathogens into the aquatic environment. Due to their filter-feeding activity, bivalves can bioaccumulate these pathogens, and because they are often consumed raw or undercooked, contaminated bivalves may pose a public health risk. Hepatitis A virus (HAV), hepatitis E virus (HEV), and norovirus genogroups I and II (NoV GI and NoV GII) are recognized by the European Food Safety Authority and the U.S. Food and Drug Administration as major etiological agents responsible for numerous foodborne outbreaks worldwide. The absence of established regulatory limits for viral contamination in food has driven the development of molecular approaches to generate scientific evidence and support risk assessment and control strategies. In this context, the present study aimed to investigate the occurrence of HAV, HEV, NoV GI, and NoV GII in commercially available mussels (*Mytilus galloprovincialis*). A total of 78 pooled samples, each comprising digestive tissues from four to five mussels, were collected from retail markets between February and December 2025 and during January and February 2026 and were analyzed by reverse transcription quantitative PCR (RT-qPCR). NoV GII RNA was detected in 5 pooled samples, corresponding to an overall pool-level positivity of 6.4% (5/78; exact 95% confidence interval [CI]: 2.1–14.3%), while HAV, HEV, and NoV GI were not detected (0/78; exact 95% CI: 0.0–4.6%). RNA concentrations in positive pooled samples were low, with a mean of 143.3 ± 132.4 copies/g, a median of 120.0 copies/g, and cycle threshold (Ct) values consistent with low-level RNA detection. Overall, the findings indicate a low but detectable pool-level positivity of NoV GII in commercially available depurated mussels. The detection of viral RNA in retail mussels indicates previous exposure to viral contamination and supports the importance of continued virus-specific surveillance of commercially available bivalves.

## 1. Introduction

Bivalve mollusks are widely used as sentinels of both chemical and microbiological contamination in marine environments. They can accumulate chemical contaminants such as cadmium (Cd), non-dioxin-like polychlorinated biphenyls (NDL-PCBs), and polycyclic aromatic hydrocarbons (PAHs) [1,2], as well as microbial hazards, including bacteria and viruses [3,4]. Their sedentary lifestyle and filter-feeding activity allow them to process large volumes of seawater, exceeding 4 L h^−1^ g^−1^ of dry tissue [5]. Consequently, contaminants present in the surrounding water can accumulate in bivalve tissues, particularly in the stomach and digestive gland [6].

Contamination of shellfish-growing areas may result from human fecal pollution originating from point-source discharges, land-based runoff, wastewater inputs, or waste disposal from vessels located near harvesting areas [7]. Because bivalve mollusks are often consumed raw or only lightly cooked, the presence of pathogens in their tissues poses a potential risk to public health [8,9]. Among commercially relevant bivalves, species in the genus *Mytilus* have been extensively investigated for their ability to harbor and accumulate a wide range of microbial contaminants [10].

Within the European Union, the hygiene and safety of live bivalve mollusks intended for human consumption are regulated through the general food hygiene requirements established by Regulation (EC) No 852/2004, the specific requirements for foods of animal origin laid down in Regulation (EC) No 853/2004, and the microbiological criteria defined in Regulation (EC) No 2073/2005. This framework primarily relies on the monitoring of fecal indicators such as *Escherichia coli*, bacterial pathogens including *Salmonella*, and marine biotoxins to assess the sanitary status of harvesting areas and the safety of products placed on the market [11,12,13]. However, routine regulatory criteria and quantitative legal limits for viral load in bivalve mollusks have not yet been established, highlighting the need for additional surveillance data to support future risk assessment and regulatory decision-making [14].

Hepatitis A virus (HAV), hepatitis E virus (HEV), and norovirus genogroups I and II (NoV GI and NoV GII) are among the enteric viruses of greatest concern in foodborne transmission [15]. Their occurrence in mussels has been documented in several studies. Diez-Valcarce et al. [16] analyzed 153 retail mussel samples and reported detection rates of 0% for HAV, 0.7% for NoV GI, and 16% for NoV GII, whereas HEV was detected in 3% of the 102 samples tested for this target. Ferri et al. [3] examined 85 pools prepared from 425 mussels and detected HAV, HEV, and NoV GI in 1.17%, 9.41%, and 2.35% of pools, respectively, while NoV GII was not detected. In a subsequent seasonal study, the same research group analyzed 355 pools comprising 1775 mussels and reported positivity rates of 0.56% for HAV, 5.35% for HEV, and 4.51% for NoV GI, with no detection of NoV GII [17]. Similarly, Fiorito et al. [18] analyzed 40 pooled samples, each prepared from the hepatopancreatic tissue of approximately 30 mussels originating from both Class A and Class B harvesting areas, and detected NoV GI and NoV GII in approximately 18% and 43% of samples, respectively; HAV and HEV were not investigated. Collectively, these findings demonstrate the variable occurrence of enteric viruses in mussels and reinforce the relevance of broader viral surveillance in shellfish production and retail chains. Against this background, the present study aimed to assess the occurrence of HAV, HEV, NoV GI, and NoV GII in retail *Mytilus galloprovincialis* marketed in Burgos, Spain, considering sampling month and commercial presentation format.

## 2. Materials and Methods

### 2.1. Sample Selection and Collection

**Figure 1:**
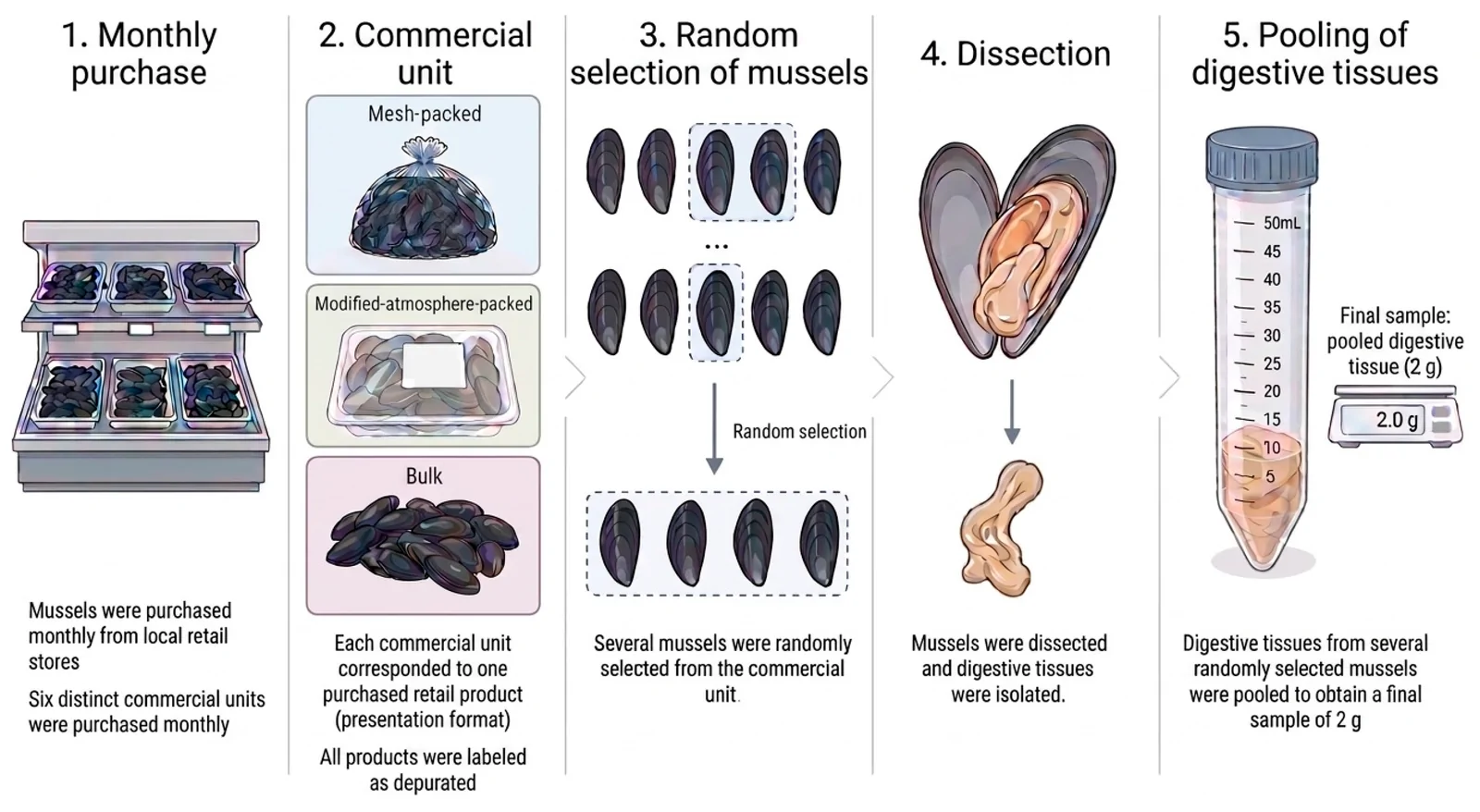
Schematic overview of mussel sampling and preparation of analytical samples. Six distinct commercial units were purchased per monthly sampling event. Each commercial unit was classified as mesh-packed, modified-atmosphere-packed, or bulk. Several mussels were randomly selected from each unit and dissected, and their digestive tissues were pooled to obtain a final analytical sample of 2 g. All commercial products were labeled as having undergone depuration before retail sales.

Mussels were purchased monthly from local retail stores in Burgos, Spain. Six distinct commercial units (mesh-packed, modified-atmosphere-packed, or bulk), each corresponding to one retail product from a different commercial brand, were obtained per monthly sampling event, resulting in a total of 78 commercial units over the study period. Each commercial unit served as the source of one pooled analytical sample. From each unit, four to five mussels were randomly selected, and their digestive tissues were pooled to obtain a 2 g analytical sample. Thus, 78 pooled analytical samples were prepared, representing approximately 312–390 individual mussels. After purchase, the samples were transported to the laboratory and processed immediately upon arrival. All samples consisted of Mediterranean mussels (*M. galloprovincialis*) originating from Galicia, Spain, and were collected monthly from February 2025 to February 2026, with the 2026 sampling period restricted to January and February. According to the information provided on the product labels, all mussels had undergone depuration before retail sales. A schematic overview of the sampling procedure and preparation of the analytical samples is shown in Figure 1.

The commercial units were obtained from five retail stores and represented nine identifiable producers. To preserve commercial confidentiality, retail stores and producers were anonymized using numerical (1–5) and alphabetical (A–I) codes, respectively. The six commercial units collected during each monthly sampling event corresponded to different commercial lots. Detailed information on the specific harvesting areas and depuration facilities was not available. Available traceability information for individual samples is provided in Table S1.

### 2.2. Virus Concentration and Nucleic Acid Extraction

Virus concentration was performed using a polyethylene glycol (PEG) 6000 (Sigma-Aldrich, Merck Life Science, Darmstadt, Germany) precipitation procedure adapted from Lewis and Metcalf (1988) [19], while the use of pooled digestive tissues followed the general approach described in UNE-EN ISO 15216-1:2017 [20]. PEG-based concentration procedures derived from the Lewis and Metcalf method have previously been applied to bivalve tissues for the recovery and detection of HAV and human noroviruses GI and GII [21], as well as HEV [22]. Bivalve shells were opened using a sterile scalpel, and the adductor muscle(s) were cut to separate the valves. The digestive tissues were dissected by removing the gills, mantle, and labial palps. Digestive tissues from four to five mussels randomly selected from each commercial unit were pooled until a total weight of 2 g was obtained, transferred to a sterile 50 mL tube, and maintained on ice (Figure 1). Mengovirus (MgV) (vMC0, Spanish Type Culture Collection [CECT], Valencia, Spain) was added as a process control (2.49 × 10^4^ Infective Units [IU]), and the pooled digestive tissues were homogenized using a manual tissue homogenizer.

For viral elution, approximately 2 g of homogenized digestive tissue was mixed with 4 mL of 10% tryptose phosphate broth (Oxoid, Basingstoke, United Kigdom) prepared in 0.05 M glycine buffer (pH 9.0) (Invitrogen, Carlsbad, CA, USA). The suspension was vortexed for 30 s (SA8 Vortex Mixer, Stuart, Bibby Sterilin Ltd., Stone, United Kingdom) and agitated at room temperature for 30 min (Luckham R300, Cole-Parmer, Vernon Hills, IL, USA). An equal volume of a chloroform–butanol solution (Sigma-Aldrich, Merck Life Science, Steinheim, Germany) (1:1, *v*/*v*) was then added. After vortexing for 30 s, the suspension was centrifuged at 8000 rpm for 15 min at 4 °C (Centrifuge 5810 R, Eppendorf SE, Hamburg, Germany), and the aqueous supernatant was transferred to a new sterile centrifuge tube.

Viral particles were precipitated by adding PEG 6000 to a final concentration of 12% (*w*/*v*). The mixture was vortexed for 30 s and continuously agitated at 4 °C for 2 h. The samples were subsequently centrifuged at 8000 rpm for 20 min at 4 °C. The supernatant was discarded, and the resulting pellet was thoroughly resuspended in 3 mL of sterile Milli-Q water (RiOs™ Essential Water Purification System, Merck Millipore, Darmstadt, Germany). Following a second centrifugation at 8000 rpm for 10 min at 4 °C, the supernatant was collected.

A 0.5 mL aliquot of the recovered supernatant was subjected to final clarification by adding 150 µL of chloroform. The mixture was vortexed and centrifuged at 8000 rpm for 10 min at 4 °C. The upper aqueous phase was carefully collected, transferred to a new sterile microcentrifuge tube, and stored at −80 °C until nucleic acid extraction and molecular analysis.

Total nucleic acids were extracted from 150 µL of each viral concentrate using the QIAamp Viral RNA Mini Kit (Qiagen, Hilden, Germany), following the manufacturer’s protocol, and eluted in a final volume of 60 µL.

Analytical performance and potential contamination were monitored using three controls in every processing batch [23,24]. Recovery efficiency was evaluated using a sample process control (SPC), prepared by inoculating 4 mL of 10% tryptose phosphate broth with an amount of MgV equivalent to that added to the samples. The SPC was subjected to all analytical steps alongside the samples and represented virus recovery in the absence of a food matrix [25]. To identify contamination introduced during sample preparation, a negative sample process control (NSPC) containing uninoculated 10% tryptose phosphate broth was processed using the same procedure. RNA extraction performance was independently monitored using an extraction control (EC), in which 10% tryptose phosphate broth was inoculated with the same quantity of MgV and introduced directly at the nucleic acid extraction stage.

The percentage of MgV recovered from each sample was estimated relative to the SPC using the following equation:

(1) $$Recovery\left(\%\right)=100\times 2^{Ct\;SPC-Ct\;Sample}$$

Recovery efficiency was assessed individually for all 78 pooled samples and was categorized as insufficient when below 5%, acceptable between 5% and 25%, good between 25% and 50%, and very good when exceeding 50%. Samples yielding less than 5% MgV recovery were considered invalid and were subjected to a new analysis beginning at the viral concentration stage, followed by nucleic acid extraction and RT-qPCR analysis. The 5% minimum recovery threshold is consistent with previous shellfish studies applying ISO-based procedures [26,27], while the recovery categories used here have precedent in previous food virology studies [28].

### 2.3. Detection of Enteric Viruses by RT-qPCR

One-step reverse transcription quantitative PCR (RT-qPCR) was used to quantify MgV and the target enteric viruses (NoV GI, NoV GII, HAV, and HEV). Each 10 µL reaction contained 2.5 µL of extracted RNA, primers, and probe at final concentrations of 0.2 µM each, and 1× TaqMan Fast Virus 1-Step Master Mix (Applied Biosystems, Waltham, MA, USA). The oligonucleotide sequences used for amplification are listed in Table S2.

Potential inhibition of reverse transcription or amplification was assessed by analyzing each RNA extract both undiluted and at a 1:10 dilution, in duplicate. Inhibition was estimated by comparing the results obtained from the undiluted and diluted extracts after correction for the dilution factor. In accordance with ISO 15216-1:2017 [20], inhibition levels of up to 75% were considered acceptable.

Quantitative synthetic nucleic acid standards obtained from American Type Culture Collection (ATCC, Manassas, VA, USA) were analyzed for each viral target in every run to monitor inter-assay performance and determine the limit of quantification (LOQ) (Table S3). Standard curve performance parameters, including slope, coefficient of determination (R^2^), and amplification efficiency for each viral target, are summarized in Table S4. The limit of quantification (LOQ) was defined as the lowest concentration of the corresponding calibration curve with a coefficient of variation (CV) ≤ 35% [29]. The limit of detection (LOD) for each viral target was experimentally assessed using serial dilutions of the corresponding standard. Each concentration was tested in three replicate RT-qPCR reactions, using 2.5 µL of template per reaction. The LOD was defined as the lowest concentration at which the target was consistently detected in all three replicate reactions.

Amplification was conducted on a QuantStudio™ 5 Real-Time PCR System (Applied Biosystems, Waltham, MA, USA), and data were processed using QuantStudio™ Design & Analysis Software v1.5.1. For HEV, reverse transcription was performed at 50 °C for 30 min, followed by an initial denaturation at 95 °C for 15 min and 45 amplification cycles of 95 °C for 10 s, 55 °C for 20 s, and 72 °C for 15 s [30]. For HAV, NoV GI, NoV GII, and MgV, reverse transcription was carried out at 55 °C for 60 min, followed by 95 °C for 5 min and 45 cycles of 95 °C for 15 s, 60 °C for 1 min, and 65 °C for 1 min [20].

A sample was classified as positive when characteristic sigmoidal amplification profiles with Ct values ≤ 40 were observed in both technical replicates, provided that no amplification occurred in the corresponding negative controls or no-template controls. This cutoff was selected because the probability of non-specific amplification increases with cycle number during RT-qPCR, resulting in a higher risk of false-positive signals at high Ct values, while amplification efficiency generally decreases at Ct values above 40, reducing assay sensitivity [31,32,33]. All NoV GII-positive pooled samples were subsequently re-analyzed in technical duplicate in a second independent RT-qPCR run using the same RNA extract.

To maintain consistent data interpretation across runs, fluorescence thresholds were manually fixed according to the software instructions. Representative amplification profiles were used to define a target-specific threshold within the initial portion of the exponential phase, approximately the first third. Because amplification curves differed among viral targets, different numerical threshold values were required to position the threshold within the same relative region of the exponential phase for each assay. Threshold values of 0.04 were applied to NoV GI, NoV GII, and HEV, whereas values of 0.08 and 0.07 were used for HAV and MgV, respectively.

### 2.4. Statistical Analysis

Viral detection (NoV GI, NoV GII, HAV, and HEV) was recorded as a binary outcome (positive or negative). For samples yielding a positive result, Ct values and viral concentrations, expressed as genome copies per gram, were also recorded.

The pool-level positivity of each viral target was calculated as the number of positive samples divided by the total number of samples analyzed. Exact 95% confidence intervals (95% CIs) for binomial proportions were estimated using the Clopper–Pearson method. Pool-level positivity was reported as the number of positive samples over the total number examined, together with the corresponding percentage and 95% CI.

Given the small number of NoV GII-positive pooled samples, subgroup analyses according to commercial presentation, sampling month, season, and year were considered exploratory. For NoV GII, which was the only viral target detected in the dataset, pool-level positivity was additionally estimated according to these variables. Seasons were defined as winter (December–February), spring (March–May), summer (June–August), and autumn (September–November). Exact 95% confidence intervals were calculated for each subgroup using the same binomial approach.

Associations between NoV GII detection and commercial presentation or season were evaluated using Fisher’s exact test. This test was selected because of the small number of positive samples and the presence of low expected frequencies in the contingency tables. Monthly findings were primarily evaluated descriptively because of the limited number of samples and positive observations within individual months.

Ct values and viral concentrations in NoV GII-positive pooled samples were summarized using the mean, standard deviation, median, minimum, and maximum. Because of the small number of positive samples, no formal comparisons of Ct values or viral concentrations among months, seasons, or presentation formats were conducted.

All statistical analyses were performed in R software v4.5.3 (R Foundation for Statistical Computing, Vienna, Austria). Statistical significance was established at *p* < 0.05.

## 3. Results

### 3.1. Occurrence of Enteric Viruses in Mussels

**Figure 2:**
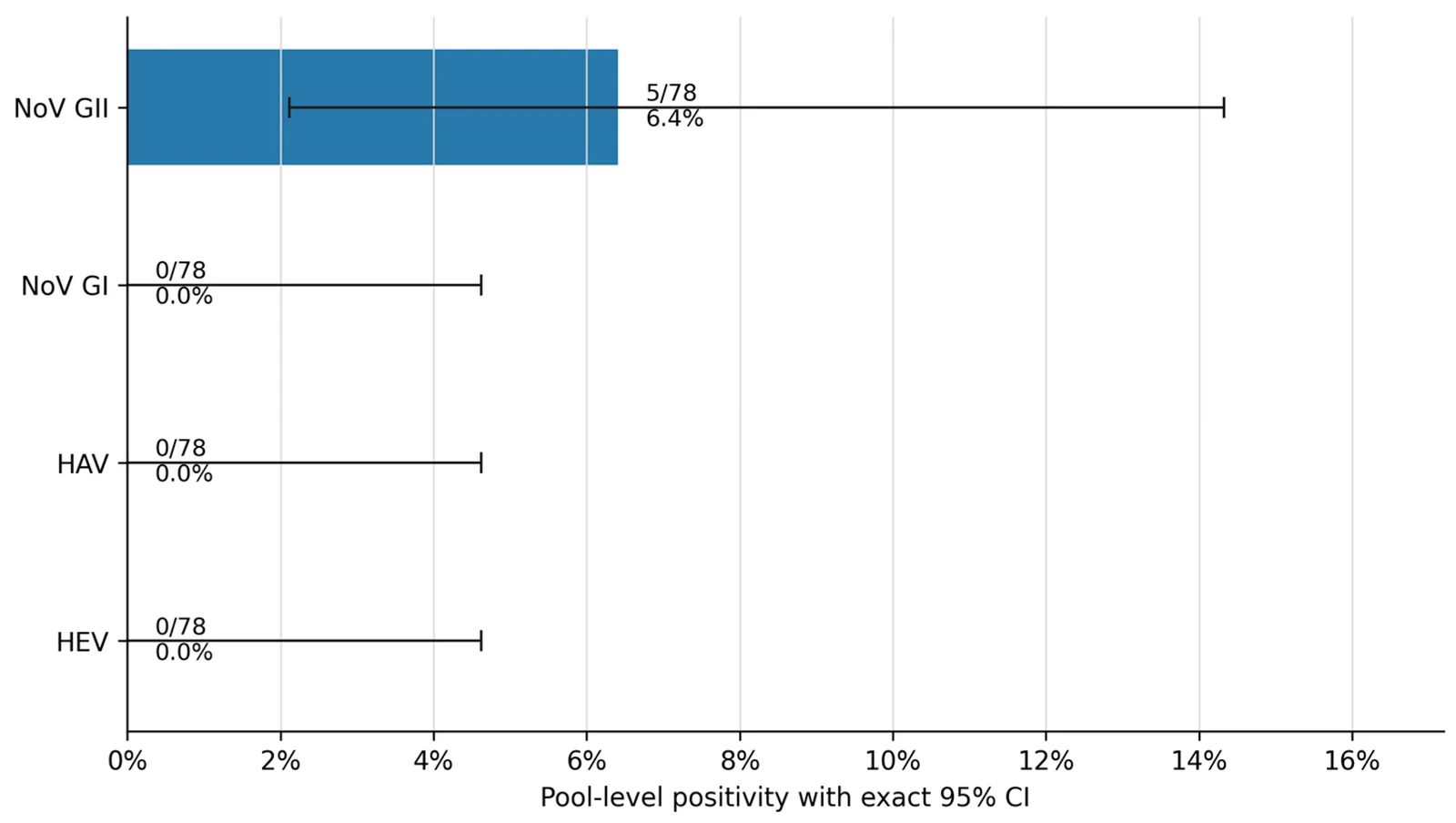
Pool-level positivity of enteric virus RNA in retail mussels (*M. galloprovincialis*) analyzed by RT-qPCR (*n* = 78). Bars represent the percentage of positive pooled samples, and error bars indicate exact 95% binomial confidence intervals. Values on the bars show the number of positive pooled samples/total samples analyzed and the corresponding pool-level positivity. NoV GI, norovirus genogroup I; NoV GII, norovirus genogroup II; HAV, hepatitis A virus; HEV, hepatitis E virus.

A total of 78 pooled mussel samples were analyzed for the presence of HEV, HAV, NoV GI, and NoV GII. HEV, HAV, and NoV GI were not detected in any of the samples, resulting in an estimated pool-level positivity of 0.0% for each viral target (0/78; exact 95% CI: 0.0–4.6%). NoV GII was detected in five pooled samples, corresponding to an overall pool-level positivity of 6.4% (5/78; exact 95% CI: 2.1–14.3%) (Figure 2).

All NoV GII-positive pooled samples were collected in 2025. One positive pooled sample was detected in April, two in May, and two in November. No positive pooled samples were identified during the remaining months. Monthly pool-level positivity was 16.7% in April (1/6; 95% CI: 0.4–64.1) and 33.3% in both May and November (2/6; 95% CI: 4.3–77.7). NoV GII was not detected in January, February, March, June, July, August, September, October, or December. The wide confidence intervals obtained for the monthly estimates reflect the limited number of samples analyzed within each month.

### 3.2. Distribution According to Season and Commercial Presentation

Given the limited number of NoV GII-positive pooled samples, the temporal and seasonal distribution of positive observations was considered descriptive and exploratory. Three of the five NoV GII-positive pooled samples were collected in spring, resulting in a seasonal pool-level positivity of 16.7% (3/18; 95% CI: 3.6–41.4). The remaining two positive pooled samples were detected in autumn, corresponding to a pool-level positivity of 11.1% (2/18; 95% CI: 1.4–34.7). NoV GII was not detected in winter (0/24; 95% CI: 0.0–14.2) or summer (0/18; 95% CI: 0.0–18.5). Although NoV GII detection was restricted to spring and autumn, the association between season and viral occurrence did not reach statistical significance (Fisher’s exact test, *p* = 0.0648).

Regarding commercial presentation, four of the five positive pooled samples were obtained from mesh-packed mussels and one from mussels packaged under modified atmosphere. NoV GII pool-level positivity was therefore 10.3% in mesh-packed samples (4/39; 95% CI: 2.9–24.2), 2.9% in modified-atmosphere-packed samples (1/34; 95% CI: 0.1–15.3), and 0.0% in bulk samples (0/5; 95% CI: 0.0–52.2). Nevertheless, no statistically significant association was observed between commercial presentation and NoV GII detection (Fisher’s exact test, *p* = 0.5471).

Pool-level positivity during the 2025 sampling period was 7.6% (5/66; 95% CI: 2.5–16.8), whereas none of the 12 samples collected in January and February 2026 were positive (0.0%; 95% CI: 0.0–26.5).

### 3.3. Analytical Performance and NoV GII RNA Concentrations in Positive Pooled Samples

The mean control virus recovery was 42.9%. Overall, 50.0% of samples showed acceptable recovery (5–25%), 21.8% showed good recovery (25–50%), and 28.2% showed very good recovery (>50%). RT-qPCR inhibition ranged from 0 to 41.8% among samples within the acceptable threshold (≤75%). Only one sample, collected in July, exceeded this threshold, showing an estimated inhibition of 99.1%. Therefore, the corresponding 1:10-diluted RNA extract was used for interpretation of the viral results. None of the 78 samples showed MgV recovery below 5%; consequently, no sample required reprocessing.

All five NoV GII-positive pooled samples were re-analyzed in technical duplicate in a second independent RT-qPCR run using the same RNA extracts. NoV GII amplification was reproduced in both technical replicates for all five samples in the second run.

The LOD was 13.75 copies/reaction for HEV and 3.45 copies/reaction for HAV, NoV GI, and NoV GII. For NoV GII, the only viral target detected in the analyzed samples, the LOQ was 8.2 genome copies/g.

Ct values in the five NoV GII-positive pooled samples ranged from 35.2 to 37.7, with a mean of 36.7 ± 1.0 and a median of 36.6. Estimated viral concentrations ranged from 48.7 to 372.2 genome copies/g, with a mean of 143.3 ± 132.4 copies/g and a median of 120.0 copies/g. Individual results are presented in Table 1 and Figure S1.

Overall, NoV GII was the only enteric virus detected and was found at a relatively low frequency and at low RNA concentrations. Positive pooled samples were identified exclusively during spring and autumn and were more frequent among mesh-packed mussels, although neither season nor commercial presentation was significantly associated with NoV GII occurrence.

## 4. Discussion

The present study provides data on the occurrence of four relevant enteric viruses at the retail level in Mediterranean mussels originating from Galicia and marketed in Burgos, Spain. This study design provides information on viral contamination in products that have passed through harvesting, depuration, processing, and distribution and are ultimately available to consumers, rather than on environmental contamination pressure or specific contamination sources, including those associated with wastewater-impacted production areas.

In the present study, NoV GII was the only viral target detected, occurring in 5 of the 78 pooled samples analyzed, corresponding to an overall pool-level positivity of 6.4%, whereas HAV, HEV, and NoV GI were not detected. Previous retail-based surveys have reported considerably variable findings. Among retail *M. galloprovincialis* originating from Galicia, NoV GII and HEV were detected in 45% and 6% of samples, respectively, whereas HAV and NoV GI were not detected [16]. NoV GI and GII were not detected in bivalves obtained from Greek seafood markets [34], while retail bivalves analyzed in Vietnam showed detection rates of 50.4% for NoV GI, 79.3% for NoV GII, 1.7% for HAV, and 11.6% for HEV [35]. HEV was detected in 2.9% of retail shellfish in Scotland [36], whereas a recent study of retail *M. galloprovincialis* from Italy reported NoV GI and GII in 77% and 40% of samples, respectively, with no detection of HAV or HEV [37]. Thus, the 6.4% NoV GII pool-level positivity observed in the present study falls within a highly heterogeneous range of findings reported at the retail level. However, these values should not be interpreted as directly comparable estimates because the studies differed in geographical origin, mollusk species, analytical unit and pooling strategy, sampling period, and analytical methodology.

Although samples collected within each monthly sampling event corresponded to different commercial lots, complete epidemiological independence cannot be assured because detailed information on harvesting areas and depuration facilities was unavailable. Among the five NoV GII-positive pooled samples, four different producers (A, C, D, and H) and three retail stores (1, 2, and 4) were represented. The two positive samples collected in May originated from different producers (C and D), retail stores (2 and 4), and commercial lots; similarly, the two positive samples collected in November originated from different producers (A and H), retail stores (1 and 2), and commercial lots. Two positive samples collected in different months (April and May) originated from the same producer (C) and retail store (2), although they corresponded to different commercial lots. Therefore, while the available traceability information supports the independence of positive commercial units collected within the same monthly sampling event, a common upstream origin cannot be completely excluded.

Consistent with the findings of the present study, norovirus, particularly NoV GII, has frequently been the predominant enteric virus detected in Spanish bivalves. Vilariño et al. [38] examined 41 samples from the Ría de Vigo, including 24 cultured and 12 wild Mediterranean mussel (*M. galloprovincialis*) samples, as well as three carpet-shell clam (*Ruditapes decussatus*) and two cockle (*Cerastoderma edule*) samples. NoV GII was detected in 53.7% of the samples, whereas NoV GI was detected in 7.3%. In the Spanish subset of Diez-Valcarce et al. [16], 51 retail samples of *M. galloprovincialis* originating from Galicia showed prevalence rates of 45% for NoV GII and 6% for HEV, while HAV and NoV GI were not detected. Manso and Romalde [39] analyzed 81 samples of *M. galloprovincialis* collected from the Ría do Burgo and detected norovirus in 49.4% of the samples, including genotypes GI.4, GII.4, and GII.6, and HAV in 18.5%. Finally, Polo et al. [40] examined 168 shellfish samples collected from ten Class B harvesting areas in two Galician rías, comprising wild and cultured *M. galloprovincialis*, clams (*Venerupis philippinarum* and *Venerupis decussata*), and cockles (*Cerastoderma edule*). In that study, NoV GI was the most prevalent target (32.1%), followed by NoV GII (25.6%) and HAV (10.1%) [40].

This pattern was also observed in studies conducted across Europe, including Italy, Slovenia, and Bulgaria. In Italy, among 59 samples of *M. galloprovincialis* and *Solen marginatus*, NoV GII was detected in 11.9% of samples, compared with 6.8% for NoV GI, while HAV was not detected [41]. In Slovenia, a study of 434 *M. galloprovincialis* samples identified NoV GII as the predominant virus during both the COVID-19 period, with prevalence rates of 15.4% for NoV GII and 0.4% for NoV GI, and the post-COVID-19 period, with rates of 35.1% and 22.9%, respectively; HAV and HEV were not detected [42]. Similarly, in Bulgaria, among 59 mussel samples, NoV GII was detected in 11.9% and NoV GI in 1.7%, whereas HAV and HEV were not detected. Notably, NoV GII prevalence reached 54.5% in samples collected from the southern coast [43].

The detection of NoV GII, but not NoV GI, may primarily reflect differences in the epidemiological circulation of the two genogroups. NoV GII generally circulates more widely in the human population and is frequently detected at higher prevalence or concentrations in wastewater. Wastewater surveillance studies conducted in Spain reported higher positivity for NoV GII than for NoV GI, including 76.0% versus 69.6% in Valencia and 83.3% versus 62.0% in Valladolid [44,45]. Although these wastewater data do not represent the specific Galician harvesting areas from which the mussels originated, they illustrate the broader predominance of NoV GII in human wastewater circulation in Spain. Greater circulation in the human population may result in higher environmental inputs of NoV GII and consequently increase the probability of exposure and detection in bivalve mollusks.

However, while the greater circulation of NoV GII may result in higher environmental exposure, the relative detection of NoV GI and GII in bivalves may also depend on differences in accumulation and persistence within their tissues. Experimental studies in oysters have shown distinct tissue-binding and bioaccumulation patterns among NoV strains; notably, GI.1 accumulated more efficiently in digestive tissues than GII.3 and GII.4, while GI.1 and GII.4 showed distinct tissue-binding patterns associated with differences in carbohydrate-ligand recognition [46,47]. Therefore, the exclusive detection of NoV GII in the present study is more plausibly related to differences in viral circulation and environmental exposure, potentially combined with strain-specific interactions with bivalve tissues, rather than to an intrinsic greater persistence or accumulation capacity of NoV GII.

Despite the predominance of NoV GII, the pool-level positivity observed in the present study was lower than that reported in previous studies, particularly those conducted in Galicia. The 78 analytical samples examined here consisted of pooled digestive tissues from four to five mussels, representing approximately 312–390 individual mussels obtained from depurated retail products. Vilariño et al. [38] analyzed 41 pooled shellfish samples, each comprising at least 10 mussels or 20 clams or cockles, and detected NoV GII in 53.7% of the samples. Their viral concentration procedure was similar to that applied in the present study, involving alkaline glycine elution, chloroform–butanol treatment, and PEG 6000 precipitation. However, their study included several bivalve species and both cultured specimens from Class B production areas and wild specimens from Class C areas, collected directly at the production stage, whereas the mussels analyzed here were retail products labeled as having undergone depuration before sale. Therefore, the lower pool-level positivity observed in the present study cannot be attributed to depuration alone and may reflect a combination of differences in sampling stage, species composition, depuration status, and analytical procedures, including variations in the viral concentration protocol.

Manso and Romalde [39] also examined mussels collected directly from harvesting areas. Their study included 81 pooled samples, each prepared from 10 mussels, representing approximately 810 individual mussels. NoV GII was detected, either alone or together with NoV GI, in 42.0% of the samples. Although they also used pooled samples, their processing procedure differed from that applied in the present study and consisted of homogenization in peptone water, low-speed centrifugation, and direct nucleic acid extraction, without PEG precipitation. Therefore, the higher pool-level positivity reported in their study cannot be attributed specifically to either the harvesting area or the viral concentration method, as both the sampling context and the analytical procedure differed between the studies. Nevertheless, contamination levels in the harvesting areas and temporal differences between the respective sampling periods may have had a greater influence than the concentration method alone.

Diez-Valcarce et al. [16] provide a closer comparison because their Spanish samples consisted of retail *M. galloprovincialis* originating from Galicia. However, the 51 mussels were processed individually rather than as pooled samples, and 45% were positive for NoV GII. In addition, Ct values of up to 45 were considered positive, whereas a Ct threshold of 40 was applied in the present study. Increasing the number of individuals included in a pool may increase the probability that the analytical unit contains at least one contaminated mussel. Conversely, pooling may dilute low concentrations of viral RNA from a single contaminated individual, potentially reducing analytical detection. Moreover, prevalence calculated at the pool level does not represent individual-mussel prevalence. Consequently, estimates based on individual mussels and pools of different sizes are not directly comparable. Overall, the lower pool-level positivity observed in the present study cannot be attributed to a single factor. In addition to differences in sample pooling and analytical criteria, the sampling context differed substantially from previous studies, as the present study analyzed commercially available mussels labeled as depurated, whereas Vilariño et al. [38] and Manso and Romalde [39] analyzed shellfish collected directly from harvesting areas. Differences in nucleic acid extraction procedures and amplification protocols among studies may also have influenced viral recovery, RNA purity, analytical sensitivity, and consequently the detection and quantification of viral RNA. Temporal variation in the circulation of NoV and in contamination pressure at the production areas may also have contributed. Furthermore, concurrent wastewater surveillance data from the specific Galician harvesting areas associated with the commercial samples were not available, preventing a direct comparison between NoV GII circulation in the source environment and its detection in retail mussels.

Although the exploratory analysis of the association between season and pool-level virus positivity was not statistically significant, several European studies have reported higher enteric virus occurrence during colder months. Lowther et al. [48] found norovirus in 90.0% of oyster samples collected in winter compared with 62.4% in summer, with the highest concentrations observed between December and March. Similar winter peaks were reported in Irish oysters by Rajko-Nenow et al. [49], in Italian mussels by Ferri et al. [17], in Slovenian mussels by Solinc et al. [42], and in bivalves from Apulia, where norovirus was detected throughout the year but occurred more frequently between December and March [50]. These observations are consistent with experimental evidence indicating that lower temperatures may enhance viral persistence, prolong retention in bivalve tissues, and slow viral elimination [51]. However, the present findings show a higher NoV GII pool-level positivity in spring. A study conducted in Galician harvesting areas reported a pattern more closely aligned with our findings, with significantly higher enteric virus prevalence between April and September [40]. Furthermore, although wastewater surveillance data specifically supporting a spring–autumn pattern in Galicia remain limited, a Spanish wastewater study, conducted in Valladolid, also identified NoV GII peaks in April and October [45]. Together, these observations suggest temporal variation in NoV GII occurrence across aquatic environments. Nevertheless, because the positive pooled samples in the present study were limited to spring and autumn, the number of detections was small, and the association with season was not statistically significant, no clear seasonal pattern can be established.

Regarding the exploratory analysis of commercial presentation, NoV GII was detected more frequently in mesh-packed mussels (4/39) than in modified-atmosphere-packed mussels (1/34), although no statistically significant association with presentation format was identified. To our knowledge, no studies have directly compared RNA stability or detection in mesh- and modified-atmosphere-packed mussels, and therefore the biological basis of this difference remains uncertain. Modified-atmosphere packaging can alter the physicochemical conditions during storage, including CO_2_ concentration, which could potentially influence RNA stability. Recent experimental evidence showed that elevated CO_2_ accelerated the decay of oyster environmental RNA in seawater [52]. However, this study evaluated non-viral environmental RNA under aquatic conditions, and its findings therefore cannot be directly extrapolated to RNA within mussel tissues under commercial modified-atmosphere packaging.

The stability of detectable NoV GII RNA may also depend on whether the genome remains protected within the viral particle or is present as free RNA. Free, unprotected NoV GII RNA has been shown to decline rapidly on food matrices, whereas RNA maintained under protective conditions remained substantially more stable [53]. Therefore, although an effect of the packaging environment on NoV GII RNA stability cannot be excluded, the present findings do not demonstrate that the lower detection frequency observed in modified-atmosphere-packed mussels resulted from CO_2_-mediated RNA degradation. The gas composition and storage conditions of the commercial products were not experimentally controlled, and the interpretation of the observed distribution is further limited by the small number of positive samples and the absence of a significant association with presentation format. Moreover, differences in production batches, harvesting areas, producers, depuration conditions, or distribution chains may also have contributed to the observed pattern.

Importantly, all mussels were labeled as having undergone depuration before retail sales, indicating that NoV GII RNA may remain detectable in commercially depurated products. Depuration of marine bivalves is an important food-safety measure based on the elimination of accumulated microorganisms when bivalves are maintained in controlled systems supplied with clean seawater [54]. Although depuration is regulated within the European Union under defined conditions to reduce microbiological contamination in live bivalve mollusks [12], the process does not guarantee the complete removal of microorganisms [54], especially enteric viruses [26,55,56,57]. For NoV specifically, persistence during depuration has been attributed, at least in part, to specific interactions between NoV particles and histo-blood group antigen (HBGA)-like carbohydrate ligands expressed in bivalve gastrointestinal tissues. Such ligands have been identified in oysters, mussels, and clams, and binding has been demonstrated for both GI and GII noroviruses [6,58]. These interactions may promote the retention of viral particles within digestive tissues and contribute to their slower elimination during depuration. Therefore, the detection of NoV GII RNA in retail mussels labeled as depurated is consistent with the recognized persistence of noroviruses in bivalve tissues. However, because information on the duration and conditions of depuration was unavailable and samples were not analyzed before and after depuration, the effectiveness of this process could not be assessed in the present study. The observed distribution may reflect differences in production batches, harvesting areas, producers, depuration conditions, or distribution chains.

Despite its contributions, this study has some limitations that should be considered. RT-qPCR detects viral RNA but does not determine viral infectivity. In addition, the high Ct values observed indicate low-level NoV GII RNA detection, consistent with findings reported in previous studies [16,18,38]. Although the detected NoV GII levels were low, the public health significance of low-level viral RNA detection remains difficult to establish. Outbreak-associated shellfish have been reported to contain relatively low NoV concentrations, including samples with fewer than 200 genome copies/g [59]. Nevertheless, outbreak-associated samples generally contain higher viral loads than non-outbreak-related shellfish, supporting an association between viral concentration and outbreak risk [59]. Therefore, low-level NoV RNA detection should not be interpreted as evidence of either negligible risk or the presence of an infectious dose [60], particularly because, as pointed out above, RT-qPCR does not distinguish infectious from non-infectious viral particles. Rather, the detection of NoV GII RNA indicates that the mussels had been exposed to viral contamination at some point in the production and distribution chain and supports continued virus-specific surveillance.

Since current European legislation does not establish mandatory criteria or quantitative limits for enteric viruses in live bivalve mollusks, virus-specific monitoring could provide baseline occurrence data, identify temporal and geographical trends, and support future risk assessment and regulatory decision-making. Overall, these findings confirm previous observations that viral genetic material may remain detectable at the retail stage and support the value of virus-specific surveillance as a complement to existing bacteriological controls.

## 5. Conclusions

NoV GII RNA was detected in 5 of the 78 pooled samples of retail Mediterranean mussels analyzed, corresponding to an overall pool-level positivity of 6.4%, whereas HAV, HEV, and NoV GI were not detected. This study provides baseline occurrence data for enteric viruses in depurated retail mussels originating from Galicia and marketed in Burgos, Spain. The results indicate sporadic, low-level NoV GII RNA detection, without evidence of a clear seasonal or commercial presentation-related pattern.

## Figures and Tables

**Table 1:** Characteristics and molecular quantification of NoV GII-positive pooled retail mussel (*M. galloprovincialis*) samples. Individual results are presented according to sampling year, commercial presentation, season, RT-qPCR Ct value, and estimated NoV GII RNA concentration. The final row summarizes the number of positive pooled samples and the median and range of Ct values and viral concentrations. Concentrations are expressed as RNA copies per gram of digestive tissue. NoV GII, norovirus genogroup II; Ct, cycle threshold.

**Sample Identification**	**Year**	**Commercial Presentation**	**Season**	**Ct Value**	**NoV GII Copies/g**
1 April 2025	2025	Mesh	Spring	37.68	48.70
1 May 2025	2025	Modified-atmosphere-packed	Spring	37.52	55.60
3 May 2025	2025	Mesh	Spring	36.58	120.00
3 November 2025	2025	Mesh	Autumn	36.59	120.01
4 November 2025	2025	Mesh	Autumn	35.21	372.22
**Number of positive pooled samples**				**Ct value, median (min–max)**	**NoV GII RNA concentration (copies/g), median (min–max)**
5				36.6 (35.2–37.7)	120.0 (48.7–372.2)

## Data Availability

The raw data supporting the findings of this article will be made available by the authors upon reasonable request.

## Supplementary Materials

The following supporting information can be downloaded at https://www.mdpi.com/article/10.3390/microorganisms14092099/s1, Figure S1: Temporal distribution of NoV GII RNA concentrations in positive pooled retail mussel samples according to commercial presentation; Table S1: Available traceability information for retail mussel samples included in the study; Table S2: Oligonucleotides and TaqMan™ probes for enteric viruses and MgV used in the study; Table S3: Exogenous standard materials obtained from ATCC; Table S4: Summary of standard curve parameters and amplification efficiency for HAV, HEV, NoV GI, and NoV GII RT-qPCR assays.

## Abbreviations

The following abbreviations are used in this manuscript:

ATCC	American Type Culture Collection
CAPES	Coordination for the Improvement of Higher Education Personnel
Cd	Cadmium
CECT	Spanish Type Culture Collection (Colección Española de Cultivos Tipo)
CI	Confidence interval
COVID-19	Coronavirus disease 2019
Ct	Cycle threshold
CV	Coefficient of variation
EC	Extraction control
HAV	Hepatitis A virus
HBGA	Histo-blood group antigen
HEV	Hepatitis E virus
IU	Infective units
LOD	Limit of detection
LOQ	Limit of quantification
MgV	Mengovirus
NDL-PCBs	Non-dioxin-like polychlorinated biphenyls
NoV	Norovirus
NoV GI	Norovirus genogroup I
NoV GII	Norovirus genogroup II
NSPC	Negative sample process control
PAHs	Polycyclic aromatic hydrocarbons
PCR	Polymerase chain reaction
PEG	Polyethylene glycol
RNA	Ribonucleic acid
RT-qPCR	Reverse transcription quantitative polymerase chain reaction
SPC	Sample process control
*v*/*v*	Volume per volume
*w*/*v*	Weight per volume
