# Supplementary material for: Occurrence of Human Enteric Viruses in Retail Mussels Marketed in Burgos, Spain
Source: Microorganisms. 2026 Sep 19;14(9):2099. doi: 10.3390/microorganisms14092099 (PMC13609700; doi:10.3390/microorganisms14092099)

Figure S1. Temporal distribution of NoV GII RNA concentrations in positive pooled retail mussel (*M. galloprovincialis*) samples according to commercial presentation. Each point represents an individual NoV GII-positive pooled sample, with marker shape and color indicating the commercial presentation format. Background shading indicates the sampling seasons. Viral concentrations are expressed as the log10-transformed number of NoV GII RNA copies per gram of digestive tissue. Only positive pooled samples are shown; no positive samples were detected among bulk mussels. NoV GII, norovirus genogroup II.

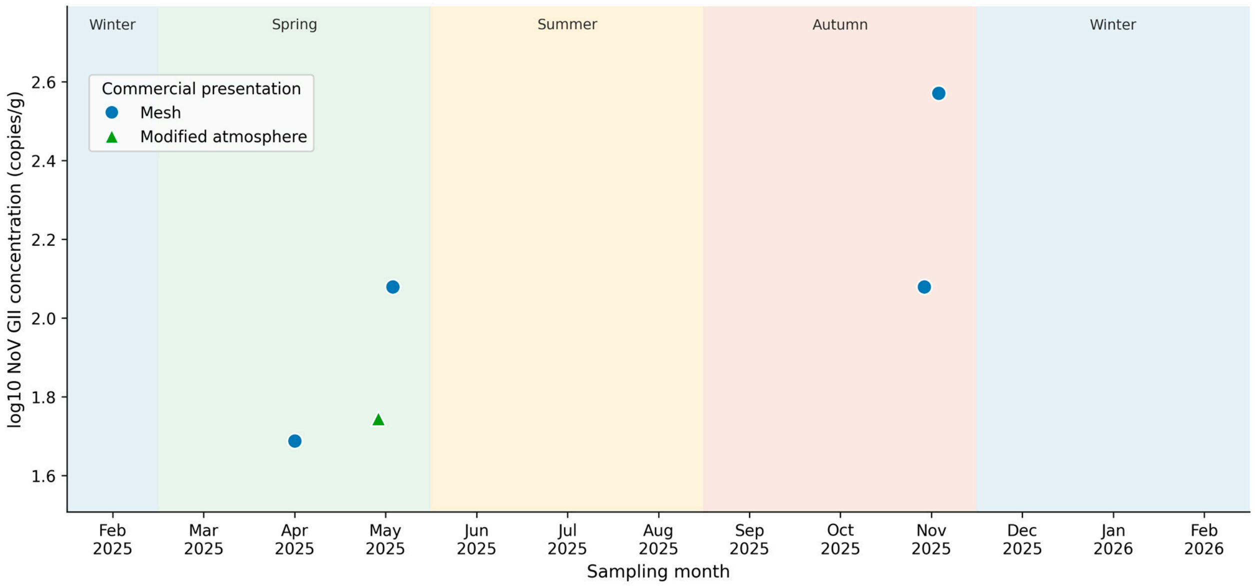

Supplement: Supplementary file 1 [file microorganisms-14-02099-s001.zip › Supplementary Material_Figures.pdf]
