# Supplementary material for: Occurrence of Human Enteric Viruses in Retail Mussels Marketed in Burgos, Spain
Source: Microorganisms. 2026 Sep 19;14(9):2099. doi: 10.3390/microorganisms14092099 (PMC13609700; doi:10.3390/microorganisms14092099)
Supplement: Supplementary file 1 [file microorganisms-14-02099-s001.zip › Supplementary Material_Tables.pdf]

Supplementary Material

Table S1. Available traceability information for retail mussel samples included in the study.

Year	Month	Sample	Producer code	Retail store code	Presentation format	Origin	Species	NoV GII result
2025	February	1	A	1	Modified atmosphere	Galicia, Spain	*Mytilus galloprovincialis*	Negative
		2	A	4	Modified atmosphere			Negative
		3	N/I	3	Modified atmosphere			Negative
		4	A	1	Mesh			Negative
		5	B	2	Mesh			Negative
		6	N/I	1	Mesh			Negative
	March	1	A	2	Modified atmosphere			Negative
		2	A	3	Modified atmosphere			Negative
		3	N/I	4	Modified atmosphere			Negative

	4	A	1	Mesh	Negative
	5	B	1	Mesh	Negative
	6	N/I	5	Mesh	Negative
April	1	C	2	Mesh	**Positive**
	2	C	2	Modified atmosphere	Negative
	3	D	4	Mesh	Negative
	4	E	1	Mesh	Negative
	5	A	1	Mesh	Negative
	6	B	5	Mesh	Negative
May	1	C	2	Modified atmosphere	**Positive**
	2	A	4	Modified atmosphere	Negative
	3	D	4	Mesh	**Positive**
	4	A	1	Mesh	Negative
	5	A	1	Mesh	Negative
	6	F	5	Mesh	Negative

June	1	A	1	Modified atmosphere	Negative
	2	G	1	Modified atmosphere	Negative
	3	C	2	Modified atmosphere	Negative
	4	A	1	Mesh	Negative
	5	A	5	Mesh	Negative
	6	N/I	5	In bulk	Negative
July	1	A	1	Modified atmosphere	Negative
	2	G	1	Modified atmosphere	Negative
	3	C	2	Modified atmosphere	Negative
	4	A	1	Mesh	Negative
	5	A	5	Mesh	Negative
	6	N/I	5	In bulk	Negative
August	1	A	1	Mesh	Negative

	2	I	4	Mesh		Negative
	3	H	1	Mesh		Negative
	4	G	1	Modified atmosphere		Negative
	5	C	2	Mesh		Negative
	6	C	2	Modified atmosphere		Negative
September	1	A	1	Mesh		Negative
	2	A	1	Modified atmosphere		Negative
	3	H	5	Mesh		Negative
	4	G	1	Modified atmosphere		Negative
	5	C	2	Modified atmosphere		Negative
	6	N/I	1	In bulk		Negative
October	1	A	1	Mesh		Negative
	2	A	1	Modified atmosphere		Negative

	3	N/I	1	In bulk	Negative
	4	G	4	Modified atmosphere	Negative
	5	C	2	Modified atmosphere	Negative
	6	C	2	Mesh	Negative
November	1	A	5	Modified atmosphere	Negative
	2	G	1	Modified atmosphere	Negative
	3	A	1	Mesh	**Positive**
	4	H	2	Mesh	**Positive**
	5	C	2	Mesh	Negative
	6	C	4	Modified atmosphere	Negative
December	1	A	1	Mesh	Negative
	2	A	4	Modified atmosphere	Negative
	3	N/I	1	In bulk	Negative

		4	A	2	Mesh		Negative
		5	C	1	Mesh		Negative
		6	C	2	Modified atmosphere		Negative
2026	January	1	A	1	Mesh		Negative
		2	A	4	Modified atmosphere		Negative
		3	G	5	Modified atmosphere		Negative
		4	I	1	Mesh		Negative
		5	H	2	Mesh		Negative
		6	C	2	Modified atmosphere		Negative
	February	1	A	4	Modified atmosphere		Negative
		2	G	1	Modified atmosphere		Negative
		3	A	1	Mesh		Negative
		4	H	1	Mesh		Negative

5	I	2	Mesh		Negative
6	C	2	Modified atmosphere		Negative

Retail stores and producers were anonymized using numerical (1–5) and alphabetical (A–I) codes, respectively. N/I indicates that producer information was not available. All samples consisted of *Mytilus galloprovincialis* originating from Galicia, Spain. The six commercial units obtained during each monthly sampling event corresponded to different commercial lots. Detailed information on specific harvesting areas and depuration facilities was not available.

Table S2. Oligonucleotides and TaqMan™ probes for enteric viruses and MgV used in the study.

Viruses	Primers	Primer and probe sequences	References
**HEV**	HEVj_FW	GGT GGT TTC TGG GGT GAC	[1]
	HEVj_RV	AGG GGT TGG TTG GAT GAA	
	HEVj_P	FAM-TGA TTC TCA GCC CTT CGC -BHQ1	
**HAV**	HAV68	TCA CCG CCG TTT GCC TAG	[2]
	HAV240	GGA GAG CCC TGG AAG AAA G	
	HAV150	FAM-CCT GAA CCT GCA GGA ATT AA-MGB	
**NoV GI**	QNIF4	CGC TGG ATG CGN TTC CAT	[2]
	NV1LCR	CCT TAG ACG CCA TCA TCA TTT AC	
	NVGG1p	FAM-TGG ACA GGA GAY CGC RAT CT-BHQ1	
**NoV GII**	QNIF2	ATG TTC AGR TGG ATG AGR TTC TCW GA	[2]
	COG2R	TCG ACG CCA TCT TCA TTC ACA	
	QNIFs	FAM-AGC ACG TGG GAG GGC GAT CG-BHQ1	
**MgV**	Mengo 110	GCG GGT CCT GCC GAA AGT	[2]
	Mengo 209	GAA GTA ACA TAT AGA CAG ACG CAC AC	

Mengo 147	FAM-ATC ACA TTA CTG GCC GAA GCMGB-BHQ1

Table S3. Exogenous standard materials obtained from ATCC.

Virus	ATCC standard material
**Hepatitis E**	Quantitative Synthetic RNA from Hepatitis E virus (ATCC VR-3258SD)
**Hepatitis A**	Quantitative Synthetic DNA from Hepatitis A virus (ATCC VR-3257SD)
**NoV GI**	Quantitative Synthetic Norovirus G1 (I) RNA (ATCC VR-3234SD)
**NoV GII**	Quantitative Synthetic RNA from Norovirus G2 (II) (ATCC VR-3235SD)

Table S4. Summary of standard curve parameters and amplification efficiency for HAV, HEV, NoV GI, and NoV GII RT-qPCR assays.

Standard curve parameter	Parameters	HAV	HEV	NoV GI	NoV GII
**Slope**	Mean	**-3.437**	**-3.506**	**-3.436**	**-3.495**
	SD	0.095	0.207	0.185	0.257
	CV	0.028	0.059	0.054	0.073
	Max	-3.344	-3.273	-3.252	-3.116
	Min	-3.546	-3.773	-3.692	-3.686
**R^2**	Mean	**0.994**	**0.988**	**0.990**	**0.997**
	SD	0.004	0.013	0.006	0.001
	CV	0.004	0.013	0.006	0.001
	Max	0.998	0.997	0.998	0.998
	Min	0.989	0.969	0.985	0.996
**Efficiency (%)**	Mean	**95.511**	**93.278**	**95.826**	**94.008**
	SD	3.596	7.407	6.817	10.354
	CV	0.038	0.079	0.071	0.110
	Max	99.087	102.083	103.003	109.375
	Min	91.429	84.095	86.576	86.766

Values represent results from five independent RT-qPCR runs.

References

1. Jothikumar, N.; Cromeans, T.L.; Robertson, B.H.; Meng, X.J.; Hill, V.R. A Broadly Reactive One-Step Real-Time RT-PCR Assay for Rapid and Sensitive Detection of Hepatitis E Virus. *J. Virol. Methods* **2006**, *131*, 65–71, doi:10.1016/j.jviromet.2005.07.004.
2. ISO 15216 ISO 15216-1:2017 - Microbiology of the Food Chain — Horizontal Method for Determination of Hepatitis A Virus and Norovirus Using Real-Time RT-PCR — Part 1: Method for Quantification Available online: https://www.iso.org/standard/65681.html (accessed on 16 July 2026).
